# A 12-Year Population-Based Analysis of Victimization and Climate Trends in Israeli Arab and Jewish Elementary Schools

**DOI:** 10.1080/15388220.2024.2385905

**Published:** 2024-09-02

**Authors:** Rami Benbenishty, Ron Avi Astor, Michal Shemesh, Dana Avital, Tal Raz, Ilan Roziner

**Affiliations:** a Hebrew University of Jerusalem, School of Social Work and Social Welfare and Universidad Andres Bello, School of Education, Santiago, Chile;; b Luskin School of Public Affairs, School of Education and Information Studies, UCLA, Los Angeles, USA;; c National Authority for Measurement & Evaluation, Ministry of Education, Ramat Gan, Israel;; d Ministry of Education, Jerusalem, Israel;; e Department of Communication Disorders, Faculty of Medical and Health Sciences, Steyer School of Health Professions, Tel Aviv, Israel

**Keywords:** School violence, bullying, time-trend, monitoring, Israel, Jewish, Arab

## Abstract

The study aims to examine changes over time in school victimization and climate in Israel, and whether these changes varied between Jewish and Arab schools and schools with different SES. A secondary analysis of the Ministry of Education database of structured student surveys regarding victimization and climate, was conducted during 2008–2019. All students in grades 5–6 were surveyed. The number of schools ranged between 751 and 1,189 (*M* = 983, *SD* = 166.3); 73.7% were Jewish schools, and 26.3% were Arab. Peer victimization dropped from 14.95 in 2008 to 7.97 in 2019 (β = −.39). All climate aspects positively increased. The highest improvements were in feeling unsafe (β = −.28). Reductions in victimization and progress in climate were the strongest among students from Arab schools and schools with lower SES. The implications for policy and future research are discussed.

School bullying is considered a global public health issue (Gimenez et al., 2024). Globally, 1 in 3 students is a victim of violence in schools (UNESCO, 2019). A vast literature has shown that both victims and perpetrators of bullying and other types of school violence experience multiple short- and long-term negative outcomes. These outcomes include emotional difficulties and impaired well-being) (Camodeca & Nava, 2022; Carretero et al., 2022); mental health difficulties such as anxiety and depression (Hong et al., 2022); school-related problems such as lower academic achievement (Gimenez et al., 2024) and increased nonattendance and dropout (Cornell et al., 2013); and higher levels of substance abuse (Pichel et al., 2022) and delinquency (Aldridge et al., 2018). The 2019 Global Burden of Disease report indicated that per 100,000 people, 26.93 disability-adjusted life years (DALYs) of anxiety disorders and 22.46 DALYs of major depressive disorder are attributable to school-based bullying victimization. The impact of experiences of school victimization and bullying on anxiety and depression has increased by about a quarter since 1990 (Hong et al., 2022).

It is important to note that much of the public discourse and scientific reports on bullying and its consequences use the term *bullying* to denote peer victimization and perpetration in school, but their definitions of bullying vary. These various definitions have been debated in recent years (UNESCO, 2024). The traditional definition requires asymmetry of strength and size between the victim and perpetrator and repeated bullying events (Olweus, 1993). However, in recent decades, many researchers and the general public have not used those concepts in their data collection and reporting. Astor and Benbenishty (2019) suggested using a more general term of school violence, encompassing any behavior, including bullying, intended to cause physical or emotional harm to individuals or groups in schools, their property, or school property. In the following sections, we use the terms *school violence* and *bullying* interchangeably to address all types of victimization, including bullying and physical, emotional, social, and cyber-based violence.

In response to the prevalence and negative impact of this problem, global awareness of school victimization and bullying as a major public health concern is growing. During the past two decades, major investments have been made to develop programs, provide resources, and enhance physical means to reduce school victimization and increase positive and safe climates in schools worldwide (Heise & Nance, 2022). It is not clear, however, whether such investments have changed the prevalence of school victimization or improved aspects of school climate that are expected to prevent school violence.

The aim of this study was to examine whether changes have occurred over time in school victimization and school climate in Israel and the extent to which these changes were similar across different groups – i.e., students from different ethnic and socioeconomic backgrounds. Identifying time trends of school victimization for various groups in the school system would provides vital knowledge about the potential impact of policies and interventions when implemented at scale and the need for additional resources, policies, and programs. For instance, there might be an increase over time in one type of school victimization (e.g., cyber-based), whereas other types are decreasing in prevalence (e.g., face-to-face victimization). This may help direct efforts to understand the reasons for the increase and use resources to target this specific type of school violence. Similarly, different social groups may vary in their levels of school victimization and school climate over time. In such cases, monitoring changes in different groups over time would help target resources for specific groups and programs to address particular victimization types, such as sexual harassment or cyber-based bullying, and focus attention on improving certain aspects of school climate, such as showing care and warmth toward students.

## Literature review of changes over time in school victimization and school climate

Many national studies have explored time trends in school victimization and bullying. These studies were conducted in a wide range of countries. One limitation of these international studies is that they used different measures and definitions, samples, and timeframes. Not surprisingly, findings from different countries have varied considerably. Some studies reported stability, others showed significant reductions in bullying and school victimization, yet others showed increases over time.

To illustrate, a New Zealand study found no change in bullying between 2001 and 2012 (Clark et al., 2013). In the United Arab Emirates, the Global School-Based Student Health Survey was employed to compare bullying victimization in 2005, 2010, and 2016 and revealed an increase in bullying (Pengpid & Peltzer, 2020). In the Czech Republic, a study comparing bullying among adolescents from 1994 to 2014 found significant decreasing trends in bullying others and being bullied, regardless of age and gender (Sarková et al., 2017).

Given the large number of studies and variations in their design and measure, it is helpful to consider reviews of these studies. Kennedy (2021) conducted meta-regression research of 91 studies of time trends in bullying and school violence. The author noted the wide range of instruments and samples employed and concluded that findings varied widely among studies. Overall, Kennedy (2021) found no significant time trend in face-to-face bullying victimization. No consistent covariates explained the variations in findings. This may be due to the vast variation in sampling and methods.

Other comprehensive reviews have explored national and international trends. Health Behaviour in School-Aged Children, a World Health Organization collaborative cross-national study, is one of the most important sources for data on health risk behaviors spanning 30 years in multiple countries. The instrument presents students with the traditional definition of bullying and asks about victimization and perpetration of bullying. The study examined temporal trends in 30 countries in Europe and North America (2001–2002, 2005–2006, and 2009–2010 surveys) and found a generally weak overall trend of a reduction in occasional bullying (from 33.5% to 29.2%) and a small reduction in chronic bullying (from 12.7% to 11.3%; Chester et al., 2015). The findings indicated that only 11 countries showed consistent trends and many countries had inconsistent trends among boys and girls. Reviewing the literature, Cosma and Associates (2017) concluded that “there is currently inconsistent evidence regarding changes over time in bullying victimization among young people” (p. 240).

Similar conclusions could be drawn from another comprehensive review of time-trend data conducted by UNESCO (2019). The review concluded that in 71 countries surveyed between 2002 and 2017, bullying had decreased in 35, increased in 13, and did not significantly change in 23. Similar changes were found for physical fights. Some differences in patterns were regional, such as consistent decreases in bullying in Western European countries and most Eastern European countries but increases or no change in almost all other English-speaking countries.

In the United States, the Centers for Disease Control and Prevention (2023) has conducted the Youth Risk Behavior Survey (YRBS) since 1991. The YRBS is the largest public health surveillance system in the United States, biannually monitoring multiple health-related behaviors, including bullying victimization, among high school students. The YRBS dashboard regarding 2009–2019 indicates that overall, the national level of bullying decreased slightly from 19.9% to 19.5% (see Li et al., 2020). An examination of the YRBS database reveals significant variations among states. For instance, whereas bullying victimization decreased by 4.5% in Texas, it increased by 8.2% in South Carolina.

The U.S. Institute of Education Science summarizes available studies and presents national trends over time in bullying and other types of school victimization. A 2023 report found major decrease in specific types of victimization and not in others: “For instance, between 2019 and 2021, the rate of nonfatal criminal victimization (including theft and violent victimization) decreased for students ages 12–18, from 30 to 7 victimizations per 1,000 students” (Irwin et al., 2023, p. 2). Student reports of being bullied at school dropped much less from 28% in 2009 to 22% in 2019.

A more recent study has not been included in prior reviews but is highly relevant to the present research (Benbenishty et al., 2023). In contrast to other studies in the United States that used small regional samples and employed the YRBS with only one item on bullying victimization, this study featured a very large representative sample of students in all secondary schools in California (*N* = 6,219,166). Assessing an 18-year trend (2001–2019), it found major reductions in multiple types of school-based victimization types and improvements in several climate indicators. The largest reduction involved being in a physical fight (from 25.4% to 11.0%), along with reductions in weapon involvement (*d* = .46) and peer victimization (*d* = .38).

In Israel, researchers have conducted several large-scale studies based on representative samples (Benbenishty & Astor, 2005). They reported on changes between three points in time: 2000, 2002, and 2005 (Benbenishty et al., 2007). Their findings indicate that whereas moderate violence dropped significantly between 2000 and 2002, no significant changes occurred between 2002 and 2005. The same pattern was reported regarding being threatened with a weapon (mostly knives) in school. In contrast, severe physical violence and sexual harassment in school were stable over time. Reductions in victimization over time were present only in Jewish schools. In Arab schools, the trend was not stable, with nonsignificant increases in moderate violence and weapon threats over time.

This study aimed to identify time trends in both school victimization and school climate. In contrast to the plethora of studies on school violence over time, we could find only two studies that examined changes over time in relevant school climate indicators. This is surprising because the importance of school climate has been well documented in the literature (Moore et al., 2020). School climate is a multidimensional construct that refers to various aspects of interpersonal interactions in the school community and school environment that are related to overall perceptions of safety, sense of engagement, and the academic and physical environment (Haynes et al., 1997; Wang & Degol, 2016). Several studies showed that school climate is associated with academic achievement, bullying, and other types of student victimization (e.g., Cornell & Huang, 2019; O’Malley et al., 2012; Steffgen et al., 2013).

Monitoring school climate is part of several educational accountability systems that assess climate over time (e.g., in Hawaii: Schweig et al., 2019; also see; Cornell & Huang, 2019). Furthermore, several studies analyzed climate longitudinal data to determine their longitudinal measurement invariance, finding that longitudinal assessment of school climate is feasible (Goulet & Morizot, 2023; Konold et al., 2021). Nonetheless, we could find only two studies that reported on school climate indicators over time (Benbenishty et al., 2023; Waasdorp et al., 2017). Waasdorp and associates asked students in a large public school district in Maryland about their belonging in school, perceptions of safety, and the degree to which adults do enough to stop bullying. During a 10-year period, there was a significant increase in safety perceptions, alongside a drop in indicators of school violence and bullying. In a representative sample of secondary schools in California, time trends over 18 years were different for the climate dimensions studied: school belongingness and safety increased (*d* = .27), adult support increased only a small amount, and student participation slightly declined during this period in California (Benbenishty et al., 2023).

This review of the literature on changes in school violence and climate suggests that changes over time are not consistent across countries and there is a need to focus on each context (see Pitsia & Mazzone, 2021).

## Context of the present study

The education system in Israel is divided into several stages. Pre-primary education consists of kindergarten for ages 3–6 and is mainly managed by local authorities. Primary education, intended for ages 6–12, begins in first grade and ends in sixth grade. Middle school caters to students aged 12–15 (Grades 7–9), whereas secondary education (high school) encompasses Grades 10–12 and is intended for students aged 15–18. The education system is centralized and managed hierarchically by the Ministry of Education.

The complexity of the Israeli society and its diverse human and cultural fabric are reflected in its education system. The system is divided into four main sectors, each with unique subsectors and characteristics: (a) national education system, which primarily serves the secular and traditional Jewish population and constitutes 39% of the student population; (b) religious national education system, aimed at the religious Jewish population, making up 14% of students, and including state-funded schools that operate according to religious lifestyles, curricula, teaching staff, and supervisors, and educate for Jewish religious values and lifestyle; (c) Arab education system, which serves the Arab, Bedouin, and Druze populations, accounting for 23% of students; and (4) ultra-Orthodox (*Haredi*) education system, intended for the ultra-Orthodox population and comprising 24% of students, a figure representative of the demographics of Israeli students. Most ultra-Orthodox schools are considered recognized but not official educational institutions, meaning they are not state-owned but comply with some compulsory education laws and assume some obligations of official education institutions. These schools have greater freedom in student admissions, teacher employment, and curriculum determination. Each sector emphasize different educational content and manages its curriculum and educational activities according to its unique values while adhering to the standards set by the Ministry of Education.

This division affects resource allocation, often resulting in budget disparities among the sectors. This is especially evident in Arab schools in poorer municipalities that provide far less supplementary resources compared to Jewish schools. Jewish religious schools tend to have lower socioeconomic status compared with Jewish secular schools. These gaps affect the quality of teaching, educational facilities, and educational opportunities available to students.

This study covers an important period in the Israeli education system- the decade following 2006. In the beginning of this century, following public sentiment that school violence was on the rise and unacceptable, and a series of studies on school violence in Israel (Benbenishty & Astor, 2005), the Israeli education system made significant changes in its priorities to allocate more resources to improve school climate and redefined the role of school counselors (Fast, 2016; Khoury-Kassabri et al., 2008). They were retrained to focus on school climate and violence prevention and center much of their professional development, duties, and resources on developing a positive school climate and coordinating the school’s efforts to prevent violence. Major investments were made in staffing and supporting counselors, especially in primary schools (Benbenishty et al., 2020). This policy is officially implemented through instructions from the general manager of the education system (https://apps.education.gov.il/mankal/horaa.aspx?siduri=492).

Parallel to efforts to improve school climate and prevent violence, the government organization in charge of evaluation and assessment (Authority for Research and Evaluation in Education, or RAMA in Hebrew) developed a mandatory monitoring system of school climate and violence (alongside academic monitoring). This system (known as *Meitzav Aklim*) provides school-level climate and violence data on each school in the system (excluding ultra-Orthodox schools) over time. In the first stages of development, the system was applied to primary schools only, then expanded to secondary schools (Astor et al., 2011). This study examined elementary school data, which cover a longer period.

## Theoretical perspective

This study is based on the theory of school victimization, bullying, and climate in evolving contexts (Astor & Benbenishty, 2019; Benbenishty & Astor, 2005). This ecological perspective addresses both the external contexts in which the school is embedded (e.g., culture, family, and neighborhood poverty) and internal contexts that the school creates (e.g., socioemotional and academic climate). In this model, school climate is important because it can buffer the influences of external contexts, such as poverty and community violence. The model differentiates among multiple victimization types (e.g., physical, emotional, relational, cyber) rather than using the general and ambiguous term of bullying. Such detail serves as an effective anchor for differential responses to diverse types of victimization.

The model puts the school in the center, rather than individual students. A focus on the school unit is essential to understand school violence and develop means to improve whole-school climate and prevent violence. Among other reasons, there is strong evidence that individual students and educators are affected by not only personally experiencing victimization but also witnessing the victimization of other students and other risky peer behaviors (Astor et al., 2006). The theoretical and practical foci are the school as a whole, and efforts are focused on preventing violence not only to benefit individual students and staff members but also to change the whole school. This school-level focus is especially relevant for research, such as the current study, that examines all schools in an education system. Finally, the model acknowledges that external and internal contexts are evolving and changing over time. It is essential, therefore, to examine schools and school systems over time.

The current study addressed many of the limitations identified in the literature. It examined a range of victimization types, rather than only bullying. This provided an opportunity to identify differential trends for different types of victimization (e.g., verbal vs. physical, moderate vs. severe). Further, it examined trends in school climate that have been studied only in a handful of studies. This study also focused on victimization trends among primary school students, who are rarely studied (for an exception, see Sourander et al., 2016). This is important because school victimization is consistently more prevalent at younger ages (Kennedy, 2021; López-Castro et al., 2023), and if victimization among primary schools decreases over time, a similar effect would be expected when these students enter secondary schools.

## Summary of aims and hypotheses

The present study overcame some limitations in previous research. It examined changes during a 12-year period in victimization types and climate indicators among almost all primary schools in Israel and identified trajectories for the overall school population and by socioeconomic and ethnocultural groups, including Jews and Arabs and Jewish secular and religious schools. Based on the theory of school victimization and climate in evolving contexts, and given contextual changes in policies and resource allocation in the Israeli educational system, our main hypotheses for the Israeli educational system were that victimization levels would decrease over time and school climate would improve over time. Given the differences in resource allocations to minority and poor schools, and the literature on this topic in Israel (Khoury-Kassabri et al., 2004) and elsewhere regarding ethnicity and poverty (e.g., Chen et al., 2024; English et al., 2024), we hypothesized that compared to others, Arab schools and schools with students with lower socioeconomic status would show less progress in preventing violence and improving school climate.

Our hypotheses were:

1. Victimization would decline.
  1a. Schools for minority students (i.e., Israeli Arab students), compared with other schools, would show a lesser decrease in victimization.
  1b. Schools with more students from lower socioeconomic backgrounds, compared with others, would show a lesser decrease in victimization.
2. School climate would improve.
  2a. Schools for minority students (i.e., Israeli Arab students), compared with other schools, would show less improvement in school climate.
  2b. Schools with more students from lower socioeconomic backgrounds, compared with others, would show less improvement in school climate.

## Method

### Design

This study involved a secondary analysis of the Ministry of Education database of student surveys conducted during 2008–2019. The surveys were carried out by an independent agency of the Ministry of Education that monitors school climate and violence and conducts annual surveys in Israeli schools. Each school is surveyed every 2 or 3 years, resulting in four to five within-school waves in this 12-year period.

### Population and sample

All public primary schools that belong to the Jewish secular and modern orthodox religious sectors and Arab schools in Israel were included. Very few ultra-Orthodox schools participate in this process, and they were not included in the study. All students in Grades 5–6 (ages 10–12) attending school were surveyed by independent proctors. The number of schools for each survey year ranged between 751 and 1,189 (*M* = 983, *SD* = 166.3, *Mdn* = 1,075). Of these, 73.7% were Jewish schools and 26.3% were Arab schools. Among Jewish schools, 65.9% belonged to the secular sector.

### Instruments

The instruments used by the monitoring system were based on scales used in multiple studies in the Israeli context (Benbenishty & Astor, 2005) and elsewhere (Green et al., 2011; López et al., 2018). The scales are presented in Arabic and Hebrew and have been used to assess school climate in Israel (with some variations over the years) since 2006.

### Independent variables

#### Ethnocultural group

Each school was identified as part of the Jewish or Arab educational system. If a school belonged to the Jewish system, it was identified as part of the Jewish secular or Jewish religious system.

#### Socioeconomic status

The Ministry of Education assesses the socioeconomic status of students’ families in each school and computes a school-level socioeconomic index (*Madad Tipuach* in Hebrew). The index features a 10-point scale. In the present study, we categorized schools as high (1–3), medium (4–6), and low (7–10) regarding socioeconomic status.

### Dependent variables

#### Victimization

Students were asked whether they were victimized in the last month (no, once or twice, three or more). The school-level index for each item was the percentage of students in school victimized at least once. The following 11 victimization items were assessed:

- A student kicked, punched, or hit me
- A student blackmailed me for money, food, or valuables
- A student gave me a serious beating
- A student pushed me
- A student threatened to hurt me in school or after school
- A student used a brick, chair, or other objects to hurt me
- A student tried to convince other students not to be my friends
- A student spread false rumors (on the internet or elsewhere) to hurt me
- A group of students boycotted me and did not want to talk or play with me
- A student cursed at me to hurt my feelings
- A student made fun of me because of my origin, religion, or the color of my skin

The overall victimization index was the mean of all items. School-level indexes were created based on aggregating student-level responses. Cronbach’s alpha for the school-level scale during the study period ranged between .82 and .87 (*Mdn* = .85).

Two items addressed students’ experiences of being victimized by a teacher: “In the last month, a teacher pushed, or shoved or hurt me on purpose” and “In the last month, a teacher made fun of me, mocked or humiliated me.”

#### Climate

Students responded to climate questions on a 5-point scale (*strongly disagree* to *strongly agree*). School-level items were computed as the percentage of students who agreed or strongly agreed regarding each item. The school-level index was the mean of items included in the scale. The following seven emotional and academic climate dimensions were assessed.

Four items were used to assess appropriate class behavior by students (e.g., “In my class, there are students who treat teachers with respect” and “In my class, there are students who are impertinent toward teachers”). Alphas ranged between .92 and .97 (*Mdn* = .95).

Regarding teacher – student caring, four items measured whether students had good relationships with teachers (e.g., “I have close and good relationships with my teachers” and “Most teachers care for me, and not only about academic issues”). Alphas ranged between .89 and .95 (*Mdn* = .92).

Three items assessed efforts to prevent violence (e.g., “Our school carries out many activities to prevent violence” and “During the break, there are teachers in the yard to prevent violence”). Alphas ranged between .81 and .86 (*Mdn* = .83).

Regarding feedback to promote learning, six items addressed corrective feedback on academic work (e.g., “When teachers return tests, they comment on what are the right answers” and “Most teachers update me on my academic status”). Alphas ranged between .96 and .98 (*Mdn* = .97).

Five items pertained to relationships among peers in class (e.g., “There is a good atmosphere among students in my class” and “Students in my class care for each other”). Alphas ranged between .83 and .93 (*Mdn* = .89).

Three items pertained to feeling unsafe (e.g., “Sometimes I’d rather stay in class during breaks because I am afraid that other students would hurt me” and “Sometimes I am afraid to go to school because students would hurt me”). Alphas ranged between .77 and .89 (*Mdn* = .85).

Three items assessed belongingness at school (“I feel good at this school,” “I love being in this school,” and “If I could, I would have changed schools”). Alphas ranged between .86 and .92 (*Mdn* = .90).

#### Analytic plan

We conducted descriptive analyses of all study variables (victimization and climate) for every year for the country overall and by economic and ethnocultural groups. We conducted regression analyses for each victimization and climate variable with year as an independent variable. We report unstandardized betas, standard errors, and standardized betas. Finally, we computed Cohen’s effect size to assess change over time. In the first stage, these analyses were conducted for the overall sample, separately for Jewish and Arab schools, and separately for Jewish religious and secular schools. In the second stage, we repeated these analyses for the three socioeconomic levels of schools (low, medium, and high).

## Findings

Hypothesis 1 posited reductions in reports of school victimization. With regard to victimization, Table 1 reports the means and standard deviations of the 11 victimization types and the index of peer victimization for every year of the study, separately for the whole sample and each ethnocultural group. The table also presents the results of the regression analysis estimating the effect of time on each victimization type. The table indicates that all types of victimization during this period showed a consistent downward trend; some reductions were very large. The overall index of peer victimization dropped from 14.95 in 2008 to 7.97 in 2019 (β = −.39, *d* = −1.59). Reductions in some types of victimization were stronger compared to others. For instance, spreading false rumors declined from 17.79 in 2008 to 9.18 in 2019 (β = −.42, *d* = −1.47) and being pushed declined from 35.25 to 20.59 (β = −.37, *d* = −1.30). Other decreases were smaller, especially in less frequent victimization types such as being blackmailed for money, food, or valuables, which dropped from 3.26 to 1.76 (β = −.11, *d* = −0.46). The prevalence of both physical and emotional victimization by teachers had meaningful reductions through the years. For instance, teachers’ emotional victimization of students dropped from 19.34 in 2008 to 12.13 in 2019 (β = −.25, *d* = −0.90). Overall, the findings in Table 1 confirm our hypothesis.

Hypothesis 1a suggested that schools for minority students (i.e., Israeli Arab students), compared with other schools, would show a lesser decrease in victimization. Table 1 presents the findings for each ethnocultural group. These findings do not support our hypotheses. In fact, for most types of victimization, reductions over time (as seen in larger beta coefficients and higher effect sizes) were stronger for Arab students. The overall index of peer victimization in Arab schools was β = −.43, compared to β = −.35 for Jewish schools. For some victimization types, the differences in time trends were especially large. For instance, the decrease among students in Arab schools in reports of blackmailing was β = −.36 (*d* = −1.04), whereas in Jewish schools it was β = −.04 (*d* = −0.23). Consistently, the gap between Arab schools and Jewish secular schools was larger than when compared to Jewish religious schools.

Regarding teacher victimization of students, the two items showed different patterns. Teachers’ physical victimization of students was much higher in Arab schools compared with Jewish schools in all years of the study. At the same time, the downward trend in teacher physical victimization in Arab schools over time (*b* = −0.71, *SE* = 0.04, β = −.34) was much stronger compared with Jewish schools (*b* = −0.29, *SE* = 0.01, β = −.23). Levels of emotional victimization by teachers, on the other hand, were similar across the study groups.

Hypothesis 1b posited that decreases in victimization would be stronger for schools with higher socioeconomic status. Table 2 presents the findings of schools based on the three levels of socioeconomic status. The findings do not confirm our hypothesis. For most types of victimization, the lowest socioeconomic status group showed the strongest decreases in victimization. To illustrate, whereas the reduction in reports of students victimized by serious beating among schools with higher socioeconomic status had an effect size of − 0.10, it was − 0.02 in medium-status schools and − 0.28 in lower-status schools.

Hypothesis 2 proposed that climate would improve throughout the 12-year period. As can be seen in Table 3, all climate aspects positively increased during the study period. The highest improvements were in feeling unsafe (*b* = −0.44, *SE* = 0.01, β = −.28), appropriate class behavior (*b* = 1.32, *SE* = 0.05, β = .26), and positive peer relations among students (*b* = 0.93, *SE* = 0.03, β = .26). Interestingly, students’ assessments that their school has tried to reduce violence showed the lowest improvement (*b* = 0.63, *SE* = 0.04, β = .14). These findings confirm Hypothesis 2.

Hypothesis 2a suggested that schools for minority students (i.e., Israeli Arab students), compared with other schools, would show less improvement in school climate. The findings in Table 3 do not confirm the hypothesis. In fact, for most indexes, the positive changes in Arab schools were larger compared with schools for other ethnocultural groups. For two indicators, the linear trends of climate improvement over time for Jewish schools were especially weak compared with those for Arab schools. Specifically, regarding efforts to reduce violence, Jewish schools underperformed (*b* = 0.42, *SE* = 0.05, β = .09) relative to Arab schools (*b* = 1.17, *SE* = 0.06, β = .35). Similarly, regarding the climate dimension of providing students with feedback to promote learning, Jewish schools made much more moderate progress (*b* = 0.41, *SE* = 0.05, β = .09) compared with Arab schools (*b* = 1.05, *SE* = 0.06, β = .29). A strong gap also existed in efforts to prevent violence: Arab schools made meaningful progress (*b* = 1.17, *SE* = 0.06, β = .35), whereas the changes in Jewish schools, especially secular schools, were much smaller (*b* = 0.23, *SE* = 0.05, β = .06).

Hypothesis 2b posited that school climate improvements would be higher in schools with higher socioeconomic status students. Table 4 presents changes over time in climate separately for the three socioeconomic groups. The pattern indicates that schools with lower socioeconomic status made bigger gains in climate. For instance, whereas lower-status schools reported the strongest gains in the appropriate class behavior indicator (*d* = 0.45), these gains were lower in medium-status (*d* = 0.32) and higher-status schools (*d* = 0.16). Changes in feeling unsafe were quite similar across the three levels of socioeconomic status (high: β = −.30, medium: β = −.34, low: β = −.33). The findings in Table 4 do not support our hypothesis.

## Discussion

This study examined data from 12 years of recurring annual surveys among elementary schools in Israel. Based on the fact that the Israeli education system invested major efforts and resources in violence prevention, Hypothesis 1 posited that there would be reductions in school violence victimization. This hypothesis was confirmed. The prevalence of all victimization types decreased over time; for some types, the reductions were very strong, including for physical and social types of victimization. For instance, the effect sizes were − 1.47 for the reduction in spreading false rumors; −2.15 for being teased because of origin, religion, or skin color; and − 1.30 for being pushed. Such reductions in victimization among primary school students in Israel are comparable to findings reported in the United States regarding secondary school students nationally (Irwin et al., 2023), in Maryland (Waasdorp et al., 2017), and in California (Benbenishty et al., 2023).

This study cannot support direct causal interpretation of the findings. Nonetheless, researchers from across the globe have tended to interpret positive gains as primarily related to efforts to prevent violence. For instance, UNESCO (2019) analyzed international trends in school violence over time and interpreted major decreases in victimization in countries such as Jamaica, Lebanon, and Uruguay as being due to major changes in commitment and policies targeting school violence, in contrast to other countries that did not show improvements. Similarly, Sarková and associates (2017) interpreted decreases in rates of bullying in the Czech Republic as indications of a political transformation that increased sensitivity and attention to school victimization. In Italy, Vieno et al. (2015) interpreted a decrease in bullying as due to the Italian government investing more systematically in prevention efforts regarding bullying. They argued that this is encouraging news for policymakers and practitioners working in the field of bullying prevention. Benbenishty and associates (2023) also made a direct connection between major reductions in victimization and resources allocated to school safety issues (e.g., Ruger, 2019) that presumably have raised awareness, increased capacity, and provided evidence-based means to address the problem. We agree with these interpretations and think there are reasons to believe that these positive changes in Israel reflect major changes in policies toward school violence prevention, including a shift in the role of counselors and the significant number of counselors added to the system to build school-level capacity, especially in elementary schools.

Our Hypothesis 2 suggested that indicators of school climate would improve over the study period. This hypothesis was confirmed. All climate dimensions examined in this study showed improvements over time. The strongest improvement was a reduction of feeling unsafe in school, with an effect size of − 0.94. Other climate aspects improved less, and their overall time trends (as captured by their beta coefficients) were not as strong as those regarding reductions in victimization.

The significant improvements in climate indicators, a prime target of the policy changes in Israel, and the fact that students noticed increased efforts over time to prevent violence in their schools add to the credibility of our interpretation that the reduction in violence is related to efforts in Israel to prevent violence through improvements in school climate. Similar changes in climate over time alongside reductions in victimization were reported in the few studies that examined trends in school climate. For instance, Waasdorp and associates (2017) reported a strong increase in students’ feelings that adults do enough to stop bullying and a significant increase in feeling safe at school. In California, there was also a strong increase in feeling safe alongside reduction in victimization and an elevated sense of belonging (Benbenishty et al., 2023).

It is important to note, however, that in these two studies, the positive changes were weaker compared to changes in victimization and these changes were not consistent across various climate dimensions. In California, there were negative changes in student participation, only small positive changes in adult support, and significant changes in belongingness (Benbenishty et al., 2023), whereas in Maryland, there was a decrease in belongingness and victimization (Waasdorp et al., 2017). These findings may reflect the complex relationships between various dimensions of school climate and school victimization. School climate may be directly associated with school violence (Astor & Benbenishty, 2019). It could also be moderating the effects of victimization on students’ outcomes. For instance, Yang and associates (Yang et al., 2018) found climate to moderate the effects of school violence on engagement. Similarly, school climate was found to moderate the relationships between bullying and academic burnout (Guo et al., 2024). A cross-lagged analysis of longitudinal data in California suggested that school climate may be an outcome, rather than an antecedent (Benbenishty et al., 2016). These complex relationships involving different indicators of school climate, school violence, and students’ outcomes over time need to be explored further.

We also hypothesized that Arab schools would show a lesser decline in victimization (Hypothesis 1a) and less improvement in climate (Hypothesis 2a) over time. This was based on our knowledge that these schools have fewer resources that could be harnessed to address these issues. These hypotheses were not supported, and Arab schools tended to show stronger reductions in victimization and improvements in school climate. There might be several reasons for this steeper decline in Arab schools. One explanation is that whereas Jewish students generally have many afterschool activities that enrich and support them, most Arab students lack such resources and are more dependent on the school. Thus, they might value its contributions more and respond more favorably to efforts to maintain peace in school. Furthermore, given the high levels of community violence, school staff members may invest more effort and personal resources in creating safe havens for their students. This interpretation may explain the fact that the climate dimension in which Arab schools made much more progress than other groups related to efforts to prevent violence.

Hypotheses 1b and 2b posited that reductions in school violence and progress in school climate would be stronger in schools with students from higher socioeconomic backgrounds. These hypotheses were not confirmed. Given that Arab schools tend to have lower socioeconomic status, these findings could be associated with the findings discussed above regarding more positive changes in Arab schools. Future studies should examine more closely schools that have lower socioeconomic status and serve different ethnocultural groups. Such an investigation may reveal interesting parallels and differences between Jewish schools (especially religious ones) and Arab schools serving populations with lower socioeconomic status.

Despite major differences in context, findings regarding changes over time in California schools show that African American students (and other minorities) reported more progress in violence reduction and school climate compared with White students (Benbenishty et al., 2023). Researchers in other countries should examine whether ethnocultural groups in their countries differ in changes to victimization and climate over time. It would be interesting to see whether gaps in resources or different cultural values and educational priorities appear to drive these differences.

### Strengths and limitations

This population-based study covered almost all schools in three of the four subsectors of the Israeli education system for more than a decade, using data gathered by a branch of the educational system that has the authority to conduct surveys on behalf of the Ministry of Education. As such, it provides representative, extensive, and reliable data. Nonetheless, some limitations should be highlighted. The study was based on aggregated school-level data and not individual data that could help explain within-school variations. A related shortcoming is that due to school confidentiality concerns, the information on each school consisted only of its ethnocultural affiliation and socioeconomic status. More school-level data (e.g., academic success, staff characteristics) would have helped identify additional sources of variability in changes over time. Further, there are no available data on secondary schools that may show different patterns than what is reported here.

### Future research

This study should be expanded and strengthened in several ways to enhance its contributions to theory, practice, and policy. Future studies should examine the relationships between school climate and violence over time. Specifically, to establish the direction of causal relationships between changes in climate and changes in victimization, researchers should explore whether changes in climate affect school violence or changes in school violence levels influence climate. It would also be helpful to add information about student outcomes to see which changes result in better psychological, behavioral, and academic performance.

This study indicates that an important area of research that needs more attention involves comparative international studies. As seen in the literature review, many differences have been found among countries, but due to significantly different methods and measurements, it is very difficult to compare and learn from these experiences. International long-term programs, such as the World Health Organization’s Health Behaviour in School-Aged Children program, should assess what country and school context factors could explain variability in violence within and between countries. Such studies would enhance the ability to learn from other countries and increase the effectiveness of efforts to prevent violence accordingly.

Finally, this study revealed the great advantages of having a national system to monitor schools over time. The current research could be conducted because of long-term systematic efforts to assess every school regarding pertinent issues such as victimization and climate. More national systems like this could make important contributions to theory, policy, and practice, both on system and school levels.

### Implications

The first and perhaps most important implication is that these findings go against the public sentiment and show that violence is not on the rise, as many believe (Sela, 2023). The findings should be encouraging to many people who are working hard to improve schools and should be shared with the public to mitigate these concerns. One reason for the need to assure the public that the situation is not out of hand is the tendency to respond to this seemingly intractable problem of youth violence by using more punitive measures, regardless of students’ rights to due process and attention to their needs and well-being.

From a policy perspective, this study provides support for policies and resources implemented in Israel to address school violence. This may also indicate to other education systems that such positive changes are possible if school-level capacity is enhanced and other important resources are provided, even in a region or country struggling with external and internal strife and conflict.

The study should draw the attention of Israeli policy makers to the uneven progress made by different groups in society. Although it may be seen as a positive sign that the groups with the fewest resources have made the strongest gains, it also should raise questions about why more progress has not been achieved by other groups. Is it an issue of lack of awareness or a deliberate process of prioritization that emphasizes other aspects of students’ lives, such as academic performance?

Finally, the study should demonstrate to policy makers the importance of a reliable and systematic monitoring system. Many politicians and educators alike have called for underemphasizing data and assessment because they think it takes away from investing in more important goals of education. In fact, in Israel, the monitoring system we describe here is under constant attack and recent changes were introduced to make monitoring more acceptable. This study should be seen as an example of how important it is to base educational and policy decisions not on intuitive impressions, dramatic events, or newspaper articles, but on data collected systematically and over long periods of time.

## Figures and Tables

**Table 1.** Means, standard deviations, betas, standard errors, and effect sizes of school victimization by cultural group and year.

	2008	2009	2010	2011	2012	2013	2014	2015	2016	2017	2018	2019			
*N*	1,088	1,058	1,107	1,093	1,149	1,121	1,185	751	798	839	790	820			
	*M* (*SD*)	*M* (*SD*)	*M* (*SD*)	*M* (*SD*)	*M* (*SD*)	*M* (*SD*)	*M* (*SD*)	*M* (*SD*)	*M* (*SD*)	*M* (*SD*)	*M* (*SD*)	*M* (*SD*)	*B* (*SE*)	β	*d*
*A student kicked, punched or hit me*															
Overall	22.26	21.62	20.10	19.69	17.36	17.37	17.94	16.96	16.12	14.97	13.75	12.69	−0.81	−.27	−1.03
	(10.20)	(10.07)	(9.62)	(10.05)	(9.23)	(9.24)	(9.79)	(9.79)	(9.63)	(8.78)	(8.71)	(8.23)	(0.03)		
Jews	23.69	23.09	21.71	20.94	18.56	18.78	18.79	18.49	17.74	16.47	15.56	14.15	−0.79	−.26	−1.03
	(10.12)	(10.08)	(9.67)	(10.01)	(9.44)	(9.45)	(10.08)	(10.08)	(9.73)	(8.92)	(8.90)	(8.28)	(0.03)		
Arabs	18.35	17.54	15.47	16.16	13.91	13.24	15.27	12.36	11.67	10.80	9.14	9.26	−0.81	−.33	−1.10
	(9.36)	(8.89)	(7.82)	(9.31)	(7.61)	(7.17)	(7.13)	(7.13)	(7.79)	(6.85)	(6.17)	(7.02)	(0.04)		
Secular	23.06	21.74	20.85	19.22	17.64	17.49	17.80	20.58	17.13	16.02	14.69	13.85	−0.74	−.33	−1.28
	(7.66)	(7.48)	(7.57)	(6.68)	(6.74)	(6.51)	(13.73)	(13.73)	(7.60)	(6.81)	(7.18)	(6.68)	(0.03)		
Religious	24.93	25.71	23.31	24.31	20.32	21.25	20.58	20.83	18.84	17.25	17.28	14.67	−0.90	−.22	−0.83
	(13.78)	(13.43)	(12.63)	(13.86)	(13.03)	(13.03)	(14.53)	(14.53)	(12.67)	(11.89)	(11.41)	(10.62)	(0.07)		
*A student blackmailed me for money, food, or valuables*															
Overall	3.26	3.10	2.85	3.21	2.63	2.53	2.99	2.53	2.54	2.41	1.87	1.76	−0.11	−.11	−0.46
	(4.12)	3.89	3.56	3.91	3.07	2.92	3.07	3.07	3.44	3.01	2.56	2.03	0.01		
Jews	1.81	1.78	1.81	2.18	1.85	1.74	2.02	1.86	1.88	1.66	1.56	1.42	−0.03	−.04	−0.23
	(1.90)	1.98	1.95	2.66	2.10	1.80	1.94	1.94	2.34	1.63	2.16	1.52	0.01		
Arabs	7.21	6.75	5.86	6.12	4.86	4.87	6.05	4.54	4.35	4.52	2.65	2.58	−0.37	−.26	−1.04
	(5.66)	5.32	5.11	5.20	4.14	4.10	4.60	4.60	4.99	4.57	3.24	2.72	0.02		
Secular Jews	1.43	1.45	1.45	1.73	1.59	1.53	1.82	2.40	1.71	1.50	1.51	1.39	0.01	.01	−0.03
	(1.54)	1.35	1.47	1.76	1.65	1.48	2.46	2.46	1.62	1.40	1.86	1.43	0.01		
Religious Jews	2.58	2.43	2.43	3.04	2.35	2.13	2.40	2.19	2.18	1.94	1.64	1.45	−0.09	−.12	−0.56
	(2.31)	2.72	2.30	3.71	2.68	2.24	2.31	2.31	3.25	1.96	2.68	1.67	0.01		
*A student gave me a serious beating*															
Overall	9.04	8.83	8.01	7.98	6.90	6.83	7.20	6.99	6.55	6.27	5.47	5.25	−0.32	−.21	−0.77
	5.80	5.84	5.12	5.41	4.74	4.52	4.89	4.89	4.57	4.46	4.09	3.91	0.01		
Jews	8.91	8.74	8.10	7.85	6.97	7.03	7.03	7.41	6.88	6.57	6.05	5.70	−0.26	−.18	−0.67
	5.54	5.81	4.97	5.15	4.61	4.49	4.82	4.82	4.31	4.29	4.16	3.93	0.01		
Arabs	9.41	9.06	7.74	8.36	6.69	6.26	7.73	5.71	5.64	5.44	3.97	4.18	−0.47	−.29	−0.99
	6.47	5.92	5.55	6.07	5.08	4.58	4.89	4.89	5.12	4.82	3.50	3.68	0.03		
Secular Jews	8.37	7.90	7.60	6.77	6.39	6.36	6.63	7.75	6.76	6.35	5.85	5.72	−0.20	−.17	−0.66
	4.49	4.10	4.27	3.28	3.90	3.43	5.84	5.84	3.59	3.57	3.80	3.50	0.01		
Religious Jews	10.02	10.40	9.03	9.94	8.09	8.30	7.75	8.53	7.09	6.96	6.45	5.65	−0.39	−.21	−0.73
	7.11	7.93	5.96	7.12	5.59	5.81	6.55	6.55	5.37	5.38	4.80	4.63	0.03		
*A student pushed me*															
Overall	35.25	34.60	31.79	31.21	27.97	28.06	27.19	25.33	24.93	23.70	21.56	20.59	−1.32	−.37	−1.30
	11.44	11.21	10.97	11.68	11.05	11.57	11.39	11.39	11.68	11.27	10.81	11.04	0.03		
Jews	37.37	36.30	33.37	32.92	28.97	29.57	28.04	27.86	27.58	26.16	24.21	22.96	−1.23	−.35	−1.33
	11.11	10.72	10.58	11.26	10.88	11.39	11.19	11.19	11.22	10.82	10.44	10.60	0.03		
Arabs	29.46	29.88	27.22	26.39	25.09	23.65	24.53	17.67	17.66	16.86	14.81	15.00	−1.49	−.44	−1.43
	10.29	11.22	10.82	11.50	11.07	10.98	8.10	8.10	9.70	9.55	8.59	10.00	0.05		
Secular Jews	37.35	35.83	33.22	31.87	28.78	29.26	28.19	27.74	28.22	26.40	24.18	23.41	−1.16	−.42	−1.59
	8.44	8.27	8.32	8.21	8.13	8.56	14.93	14.93	8.81	8.79	8.70	9.05	0.03		
Religious Jews	37.35	37.22	33.57	34.95	29.32	30.16	27.74	28.14	26.40	25.55	24.27	22.13	−1.37	.30	−1.08
	15.15	14.31	13.96	15.41	14.84	15.42	15.98	15.98	14.55	13.78	13.36	12.97	0.08		
*A student threatened to hurt me in school or after school*															
Overall	12.36	12.10	11.31	11.30	9.04	8.54	8.65	7.66	7.08	6.89	5.75	5.29	−0.67	−.35	−1.22
	7.22	7.46	6.66	6.90	6.00	5.74	5.19	5.19	5.17	4.95	4.20	3.87	0.02		
Jews	10.90	10.62	10.30	10.26	8.02	7.66	7.50	6.99	6.37	6.25	5.70	4.87	−0.57	−.34	−1.24
	5.96	6.24	5.78	6.11	4.98	4.98	4.57	4.57	4.39	4.18	4.24	3.40	0.02		
Arabs	16.32	16.20	14.23	14.23	11.99	11.15	12.29	9.69	9.03	8.66	5.87	6.26	−0.95	−.41	−1.44
	8.72	8.93	8.03	8.08	7.53	6.91	6.31	6.31	6.50	6.31	4.11	4.66	0.04		
Secular Jews	10.35	9.81	9.95	9.28	7.75	7.21	7.16	8.10	6.30	6.11	5.46	5.01	−0.51	−.37	−1.28
	5.00	4.85	5.21	4.59	4.33	3.98	6.19	6.19	3.87	3.42	3.68	3.11	0.02		
Religious Jews	12.00	12.20	10.93	12.19	8.52	8.50	8.10	7.76	6.50	6.51	6.17	4.60	−0.70	−.34	−1.25
	7.45	8.08	6.69	7.98	6.02	6.40	6.29	6.29	5.21	5.32	5.18	3.85	0.04		
*A student used a brick, chair, or other objects to hurt me*															
Overall	7.49	7.49	6.92	7.32	6.07	6.07	6.25	5.96	5.48	5.03	4.06	3.85	−0.32	−.22	−0.79
	5.67	5.68	4.99	5.51	4.83	4.50	4.69	4.69	4.49	4.31	3.81	3.24	0.01		
Jews	6.32	6.46	6.14	6.50	5.44	5.55	5.52	5.72	5.25	4.62	4.24	3.79	−0.22	−.17	−0.68
	4.35	4.86	4.22	4.61	4.03	3.98	4.53	4.53	3.99	3.54	3.93	3.00	0.01		
Arabs	10.67	10.35	9.20	9.63	7.90	7.58	8.57	6.65	6.13	6.17	3.59	4.01	−0.61	−.33	−1.14
	7.39	6.73	6.19	6.99	6.28	5.50	5.08	5.08	5.61	5.80	3.45	3.75	0.03		
Secular Jews	5.63	5.55	5.43	5.60	4.97	5.09	5.09	6.28	4.92	4.43	4.00	3.87	−0.15	−.15	−0.59
	3.15	3.29	3.16	3.10	3.06	2.99	5.58	5.58	3.17	2.86	3.56	2.78	0.01		
Religious Jews	7.74	8.21	7.47	8.25	6.35	6.43	6.28	6.60	5.84	4.90	4.72	3.63	−0.37	−.22	−0.86
	5.86	6.62	5.49	6.29	5.33	5.28	5.92	5.92	5.12	4.39	4.57	3.37	0.03		
*A student tried to convince other students not to be my friends*															
Overall	18.28	17.89	16.37	16.56	14.26	13.66	13.45	11.95	11.96	11.05	9.73	9.34	−0.84	−.36	−1.25
	8.40	8.22	7.83	8.24	7.45	7.41	6.25	6.25	6.46	6.40	5.63	5.60	0.02		
Jews	16.83	16.30	14.96	14.51	12.40	11.94	11.37	10.51	10.54	9.67	9.16	8.46	−0.78	−.40	−1.38
	7.34	7.14	6.91	6.27	5.71	5.93	4.98	4.98	5.04	4.86	4.99	4.47	0.02		
Arabs	22.24	22.32	20.44	22.35	19.62	18.69	20.01	16.29	15.87	14.88	11.17	11.42	−1.03	−.37	−1.26
	9.73	9.35	8.84	10.19	9.09	8.90	7.58	7.58	8.11	8.33	6.80	7.23	0.05		
Secular Jews	16.87	16.42	14.91	14.67	12.67	12.40	11.73	10.68	11.27	9.93	9.94	9.24	−0.71	−.38	−1.30
	7.04	6.69	6.78	5.64	5.54	5.68	6.27	6.27	4.72	4.63	4.71	4.37	0.02		
Religious Jews	16.74	16.05	14.99	14.21	11.88	11.06	10.68	9.38	9.20	9.04	7.58	7.01	−0.91	−.43	−1.52
	7.94	7.95	7.14	7.35	6.03	6.29	5.31	5.31	5.32	5.04	5.20	4.30	0.03		
*A student spread false rumors (on the internet or elsewhere) to hurt me*															
Overall	17.79	17.19	16.91	16.11	13.36	12.57	11.96	11.07	11.03	10.74	9.44	9.18	−0.85	−.42	−1.47
	6.47	6.60	8.16	6.44	5.84	5.79	5.08	5.08	5.61	5.40	5.41	5.19	0.02		
Jews	18.20	18.10	17.19	16.42	13.45	12.75	11.69	11.45	11.56	11.21	10.36	10.01	−0.83	−.44	−1.45
	6.30	6.35	6.33	5.96	5.64	5.49	4.77	4.77	5.10	4.99	5.11	4.87	0.02		
Arabs	16.67	14.65	16.08	15.23	13.10	12.04	12.80	9.95	9.57	9.44	7.09	7.21	−0.89	−.39	−1.54
	6.80	6.63	11.94	7.57	6.39	6.55	5.81	5.81	6.60	6.21	5.46	5.38	0.04		
Secular Jews	18.49	18.84	17.63	16.92	14.33	13.69	12.44	10.28	12.69	11.95	11.48	11.20	−0.77	−.43	−1.40
	5.59	5.93	6.22	5.49	5.48	5.20	5.37	5.37	4.88	4.71	4.94	4.77	0.02		
Religious Jews	17.58	16.66	16.28	15.43	11.71	10.97	10.28	9.71	9.53	9.78	8.15	7.85	−0.94	−.47	−1.59
	7.54	6.89	6.41	6.69	5.55	5.60	5.15	5.15	4.88	5.19	4.73	4.30	0.03		
*A group of students boycotted me and did not want to talk or play with me*															
Overall	8.82	8.73	7.85	8.02	6.79	6.39	6.53	5.63	5.52	5.01	3.93	3.74	−0.47	−.26	−0.91
	7.03	6.97	6.28	6.95	5.95	5.82	5.01	5.01	5.02	5.11	3.85	3.67	0.02		
Jews	6.42	6.39	5.69	5.44	4.57	4.27	4.28	3.84	3.77	3.32	2.94	2.69	−0.35	−.33	−1.05
	4.57	4.15	3.81	3.72	3.19	3.07	2.71	2.71	2.43	2.43	2.74	2.10	0.11		
Arabs	15.35	15.24	14.07	15.32	13.19	12.60	13.60	11.02	10.32	9.68	6.45	6.23	−0.83	−.36	−1.32
	8.31	8.85	7.70	8.58	7.27	7.35	6.33	6.33	6.85	7.26	4.97	5.10	0.04		
Secular Jews	6.21	6.16	5.43	5.14	4.56	4.21	4.30	4.22	3.98	3.30	3.10	2.93	−0.30	−.31	−1.00
	4.10	3.59	3.61	3.03	3.11	2.84	3.04	3.04	2.40	2.30	2.26	2.17	0.01		
Religious Jews	6.87	6.83	6.14	6.02	4.58	4.39	4.22	3.74	3.40	3.32	2.61	2.24	−0.44	−.36	−1.15
	5.39	5.04	4.04	4.75	3.35	3.48	3.22	3.22	2.46	2.60	3.51	1.91	0.02		
*A student cursed at me to hurt my feelings*															
Overall	–	34.43	32.34	31.65	25.64	25.46	25.29	25.05	23.98	22.96	20.25	19.76	−1.39	−.34	−2.51
		13.51	12.50	12.69	11.13	11.38	11.73	11.73	12.17	11.67	10.98	11.14	0.04		
Jews	–	39.14	35.65	35.86	28.46	28.66	28.04	28.63	27.72	26.40	23.89	23.28	−1.44	−.38	−3.22
	–	11.01	11.16	10.63	10.26	10.37	10.50	10.50	10.92	10.56	9.59	10.23	0.04		
Arabs	–	21.33	22.78	19.77	17.51	16.07	16.62	14.26	13.72	13.38	11.02	11.46	−1.14	−.36	−1.92
	–	10.95	11.18	10.30	9.43	8.71	8.09	8.09	9.17	8.93	8.62	8.46	0.06		
Secular Jews	–	39.99	36.68	36.08	29.12	29.02	28.80	26.55	29.18	27.32	24.46	24.85	−1.40	−.44	−4.00
	–	8.54	9.78	8.23	8.39	8.24	14.07	14.07	8.86	8.54	8.12	8.78	0.04		
Religious Jews	–	37.49	33.61	35.42	27.18	27.97	26.55	27.26	25.07	24.57	22.84	20.43	−1.52	−.33	−2.41
	–	14.57	13.23	14.20	13.09	13.54	13.68	13.68	13.52	13.39	11.96	12.00	0.08		
*A student made fun of me because of my origin, religion or the color of my skin*															
Overall	–	15.00	15.59	15.18	12.25	12.07	11.53	11.21	10.62	10.09	8.76	8.45	−0.73	−.32	−2.15
	–	7.22	8.30	7.43	6.65	6.29	6.29	6.29	6.40	6.12	5.83	5.57	0.02		
Jews	–	15.56	15.03	15.86	12.69	12.54	11.58	11.90	11.24	10.82	9.84	9.48	−0.64	−.29	−2.39
	–	7.29	7.02	7.45	6.58	6.30	6.41	6.41	6.43	5.82	6.01	5.62	0.02		
Arabs	–	13.45	17.18	13.26	11.01	10.68	11.39	9.13	8.94	8.06	6.03	6.04	−0.93	−.39	−1.84
	–	6.78	11.07	7.03	6.69	6.08	5.45	5.45	6.03	6.47	4.27	4.65	0.04		
Secular Jews	–	14.11	13.91	14.33	11.67	11.56	10.40	13.77	10.42	9.96	8.92	8.77	−1.13	−.35	−2.83
	–	5.54	5.84	5.67	5.34	5.21	7.88	7.88	5.21	4.86	4.70	4.38	0.06		
Religious Jews	–	18.39	17.18	18.83	14.67	14.40	13.77	13.95	12.72	12.44	11.68	10.74	−0.58	−.32	−2.11
		9.22	8.44	9.38	8.15	7.64	7.94	7.94	7.99	7.04	7.73	7.19	0.02		
*Index of peer victimization*															
Overall	14.95	14.62	13.57	13.49	11.60	11.34	11.35	10.45	10.13	9.56	8.39	7.97	−0.63	−.39	−1.46
	5.52	5.56	5.14	5.60	4.90	4.89	5.03	4.62	4.71	4.51	4.17	3.94	0.01		
Jews	14.50	14.20	13.25	13.00	11.14	11.03	10.69	10.46	10.17	9.55	8.87	8.23	−0.55	−.35	−1.43
	4.98	5.01	4.65	4.85	4.29	4.40	4.55	4.36	4.26	4.03	4.10	3.72	0.02		
Arabs	16.19	15.78	14.48	14.87	12.93	12.23	13.43	10.43	10.03	9.60	7.19	7.35	−0.84	−.43	−1.58
	6.63	6.73	6.25	7.14	6.15	6.03	5.85	5.35	5.80	5.64	4.11	4.36	0.03		
Secular Jews	14.20	13.75	12.94	12.36	10.96	10.80	10.57	10.30	10.33	9.55	8.91	8.51	−0.50	−.39	−1.51
	4.11	3.93	4.04	3.54	3.51	3.52	3.74	3.24	3.53	3.41	3.63	3.39	0.02		
Religious Jews	15.09	15.08	13.79	14.26	11.46	11.47	10.89	10.76	9.89	9.47	8.76	7.69	−0.67	−.33	−1.37
	6.38	6.55	5.57	6.54	5.50	5.71	5.74	6.05	5.33	4.97	4.94	4.22	0.03		
*Teacher physical victimization of students*															
Overall	7.70	7.21	6.58	7.62	5.89	5.74	5.99	4.87	4.93	4.35	3.52	3.15	−0.40	−.23	−0.85
	6.77	6.50	5.74	6.79	5.68	5.93	5.76	4.89	4.95	4.71	3.83	3.43	0.02		
Jews	5.73	5.12	4.93	5.76	4.40	3.97	4.24	3.47	3.82	3.01	2.85	2.26	−0.29	−.23	−0.90
	4.96	4.43	4.28	5.36	4.33	4.25	4.21	3.33	3.60	2.98	3.22	2.28	0.01		
Arabs	13.07	13.01	11.32	12.86	10.17	10.93	11.47	9.10	7.99	8.08	5.21	5.25	−0.71	−.34	−1.20
	8.02	7.73	6.70	7.62	6.84	7.00	6.48	6.24	6.60	6.37	4.67	4.60	0.04		
Secular Jews	4.59	3.93	4.00	4.70	3.73	3.22	3.62	2.86	3.40	2.60	2.42	2.03	−0.21	−.23	−0.92
	3.52	2.73	3.00	3.64	3.02	2.68	3.48	2.47	2.74	2.41	2.58	1.76	0.01		
Religious Jews	8.05	7.43	6.70	7.83	5.71	5.41	5.38	4.71	4.59	3.78	3.73	2.66	−0.46	−.27	−1.07
	6.45	5.93	5.60	7.25	5.92	5.99	5.11	4.38	4.70	3.73	4.11	2.96	0.03		
*Teacher emotional victimization of students*															
Overall	19.34	18.43	17.72	18.41	15.70	15.39	16.06	15.60	15.30	14.27	12.55	12.13	−0.60	−.25	−0.91
	8.37	7.70	7.49	8.30	7.70	7.64	7.86	7.33	8.11	7.97	7.46	7.37	0.02		
Jews	19.24	18.16	17.81	18.09	15.25	14.94	15.52	15.77	15.93	14.58	13.44	13.24	−0.49	−.21	−0.76
	8.36	7.61	7.34	7.89	7.31	7.23	7.66	7.15	7.88	7.69	7.23	7.35	0.02		
Arabs	19.60	19.17	17.47	19.30	17.01	16.69	17.76	15.07	13.58	13.41	10.31	9.49	−0.87	−.34	−1.33
	8.41	7.90	7.95	9.34	8.62	8.60	8.24	7.87	8.50	8.65	7.58	6.72	0.04		
Secular Jews	17.63	16.31	16.41	17.01	14.38	14.31	14.98	15.48	15.81	14.29	13.08	13.32	−0.33	−.16	−0.60
	7.43	6.49	6.60	7.18	6.74	6.79	6.91	6.61	7.22	7.33	6.54	6.97	0.03		
Religious Jews	22.48	21.77	20.41	20.20	16.95	16.16	16.52	16.32	16.14	15.07	14.21	13.08	−0.82	−.31	−1.09
	9.17	8.32	7.87	8.75	8.06	7.89	8.83	8.13	8.98	8.29	8.44	8.04	0.05		

**Note**. Missing values indicate that item was not asked that year.

**Table 2.** Means, standard deviations, betas, standard errors, and effect sizes of school victimization by SES and Year.

	2008	2009	2010	2011	2012	2013	2014	2015	2016	2017	2018	2019			
	1,088	1,058	1,107	1,093	1,149	1,121	1,185	751	798	839	790	820			
*N*	*M* (*SD*)	*M* (*SD*)	*M* (*SD*)	*M* (*SD*)	*M* (*SD*)	*M* (*SD*)	*M* (*SD*)	*M* (*SD*)	*M* (*SD*)	*M* (*SD*)	*M* (*SD*)	*M* (*SD*)	*B* (*SE*)	β	*d*
*A teacher hit or shoved me*															
High	4.15	3.8	3.85	4.59	3.43	2.96	3.12	2.67	3.09	2.55	2.21	2.21	−0.38	−.19	−0.13
	(3.47)	3.09	3.45	3.50	2.78	2.64	2.81	2.40	2.57	2.3	1.76	1.87	0.04		
Medium	6.83	6	5.97	6.36	5.12	4.82	5.31	4.28	4.64	3.39	3.19	2.73	−0.59	−.24	−0.21
	(5.57)	4.89	4.95	5.38	4.61	4.88	4.69	3.92	4.80	3.41	3.35	3.06	0.03		
Low	11.56	11.58	9.57	11.61	8.74	9.34	9.29	7.57	6.98	7.28	5.17	4.60	−0.82	−.32	−0.30
	(7.85)	7.73	6.77	8.28	7.04	7.24	7.16	6.23	5.96	6.26	5.06	4.44	0.04		
*A teacher mocked, insulted, or humiliated me in school*															
High	19.35	18.67	18.29	19.03	15.87	15.79	16.19	16.14	17.39	16.2	14.21	15.26	−0.19	−.22	−0.15
	7.36	6.55	6.71	6.67	6.55	6.29	7.27	6.15	7.52	7.11	6.44	6.47	0.01		
Medium	19.44	17.87	17.84	17.74	15.35	14.76	15.68	15.43	15.33	13.96	12.94	11.73	−0.34	−.24	−0.26
	8.71	7.99	7.34	7.98	7.68	7.71	7.89	7.98	8.16	8.04	8.17	7.53	0.02		
Low	19.44	18.79	17.2	18.51	15.98	15.81	16.53	15.3	13.34	13.02	10.51	9.71	−0.61	−.28	−0.34
	8.45	8.22	8.08	9.57	8.23	8.52	8.25	7.51	8.12	8.31	7.02	6.91	0.03		
*A student kicked, punched, or hit me*															
High	22.54	21.16	20.33	19.3	16.99	17.31	17.01	16.87	16.28	15.33	14.73	14.08	−0.71	−.28	−0.29
	8.43	8.67	7.79	8.81	7.54	8.71	8.33	8.45	8.22	8.04	7.58	6.94	0.04		
Medium	22.54	22.73	20.73	20.58	17.41	18.69	18.62	17.87	17.76	16.05	14.56	13.65	−0.75	−.24	−0.27
	11.22	10.64	10.65	10.32	10.21	9.81	10.41	10.9	10.63	9.42	9.17	9.03	0.04		
Low	21.42	20.37	19.15	18.65	17.46	15.67	17.97	15.95	13.96	13.22	11.86	10.11	−0.93	−.32	−0.37
	9.72	9.86	9.37	10.11	8.76	8.51	9.49	9.39	9.16	8.19	8.9	7.64	0.05		
*A student blackmailed me for money, food, or valuables*															
High	1.44	1.48	1.61	1.81	1.56	1.44	1.72	1.57	1.51	1.58	1.32	1.32	−0.01	−.03	−0.01
	1.47	1.39	1.39	1.63	1.59	1.28	1.72	1.36	1.28	1.41	1.11	1.21	0.01		
Medium	2.61	2.14	2.39	2.49	2.09	2.05	2.46	2.06	2.21	1.73	1.47	1.6	−0.08	−.11	−0.07
	2.61	2.26	2.65	3.05	2.29	2.01	2.53	2	2.7	1.78	1.72	1.86	0.01		
Low	5.66	5.82	4.53	5.28	4.1	4.14	4.78	3.97	3.91	4.04	2.86	2.39	−0.25	−.18	−0.18
	5.85	5.46	4.99	5.04	3.95	4.07	4.92	4.43	4.94	4.35	3.81	2.63	0.02		
*A student gave me a serious beating*															
High	8.04	7.64	7.54	6.94	6.17	6.45	6.33	6.81	6.31	6.17	5.72	5.93	−0.18	−.16	−0.10
	4.09	4.03	3.96	3.79	3.39	3.9	3.67	4.17	3.7	3.8	3.24	3.51	0.02		
Medium	8.54	8.79	7.9	7.85	6.65	7.07	7	7.2	6.92	6.35	5.47	5.36	−0.27	−.18	−0.15
	5.61	6.43	5.18	5.55	4.51	4.59	4.6	5.25	4.85	4.48	4.14	4.04	0.02		
Low	10.52	9.94	8.55	8.82	7.56	6.84	8.29	6.91	6.29	6.27	5.22	4.44	−0.48	−.28	−0.28
	6.81	6.04	5.61	5.96	5.05	4.87	5.72	5.06	4.9	4.96	4.72	3.97	0.03		
*A student pushed me*															
High	37.49	36.03	33.4	32.32	28.07	29.4	27.79	27.53	27.62	26.48	24.48	24.38	−1.14	−.36	−0.40
	9.61	10.31	8.87	10.06	9.22	10.6	9.9	9.99	9.46	10.11	9.28	9.1	0.05		
Medium	35.76	35.3	32.2	32.42	27.92	29.07	27.23	26.15	26.32	24.6	22.45	21.25	−1.28	−.34	−0.39
	12.53	11.2	11.57	11.84	11.66	11.66	11.37	12.08	12.43	11.72	11.05	11.42	0.05		
Low	32.6	32.27	29.97	28.75	27.8	25.64	26.84	22.42	20.8	19.99	17.7	16.15	−1.51	−.41	−0.46
	10.81	11.43	11.22	11.99	11.48	11.63	11.04	11.19	11.43	10.69	10.82	10.68	0.06		
*A student threatened to hurt me in school or after school*															
High	9.1	9.03	8.94	8.47	6.44	6.34	6.11	5.98	5.16	5.22	4.97	4.59	−0.46	−.37	−0.25
	4.25	4.78	4.49	4.6	3.4	4.08	3.98	3.64	3.24	3.38	3.19	2.76	0.02		
Medium	11.47	11.27	10.81	10.96	8.12	8.16	8.15	7.34	7.1	6.48	5.46	4.95	−0.61	−.36	−0.32
	6.39	6.64	5.98	6.22	5	5	5.12	5	4.9	4.3	3.86	3.43	0.02		
Low	16.24	15.85	13.75	13.99	12.24	11.12	11.52	9.56	8.83	8.94	6.83	6.39	−0.9	−.39	−0.40
	8.45	8.48	7.94	7.76	7.29	6.83	7	5.96	6.29	6.12	5.14	4.95	0.03		
*A student used a brick, chair, or other objects to hurt me*															
High	5.44	5.29	5.49	5.49	4.63	4.81	4.68	4.83	4.52	4.16	3.63	3.87	−0.16	−.17	−0.09
	3.19	3.41	3.64	3.56	2.98	3.28	3.32	3.48	3.26	2.85	2.58	2.82	0.02		
Medium	6.66	6.85	6.37	6.79	5.41	5.78	5.61	5.66	5.56	4.46	3.92	3.71	−0.27	−.2	−0.15
	4.69	5	4.24	4.97	4.12	4.03	4.19	4.44	4.58	3.52	3.65	3.38	0.02		
Low	10.14	10.29	8.67	9.42	8.1	7.51	8.45	7.31	6.32	6.57	4.63	4.03	−0.51	−.28	−0.30
	7.09	6.84	6.09	6.42	6.02	5.25	6.47	5.51	5.19	5.74	4.81	3.42	0.03		
*A student tried to convince other students not to be my friends*															
High	16.58	15.55	14.24	13.55	11.7	11.24	10.73	9.61	10.23	9.19	9.11	8.95	−0.71	−.43	−0.35
	6.17	6.19	6.02	4.93	4.96	4.98	4.52	3.8	3.92	3.69	4.4	4.02	0.03		
Medium	17.12	16.93	15.66	15.64	13.25	12.68	12.32	11.56	11.3	10.3	9.48	8.8	−0.78	−.37	−0.32
	7.85	7.61	7.14	7.34	6.72	6.84	6.44	5.93	6.27	5.75	5.67	5.47	0.03		
Low	21.5	21.26	19.2	20.48	17.76	17.25	17.36	14.55	14.42	13.72	10.62	10.44	−1.02	−.37	−0.38
	9.49	9.15	9.13	9.9	8.63	8.54	8.53	7.37	7.76	8.09	6.48	6.79	0.04		
*A student spread false rumors (on the internet or elsewhere) to hurt me*															
High	18.81	18.91	17.56	16.39	13.72	13.07	11.85	11.66	11.91	11.65	10.82	11.31	−0.8	−.47	−0.33
	5.35	5.91	6.02	5.08	5.21	4.91	4.89	4.2	4.63	4.54	4.69	4.31	0.03		
Medium	17.1	17.02	17.04	15.94	12.64	12.31	11.25	10.89	11.01	10.54	9.52	9.14	−0.82	−.40	−0.32
	6.28	6.58	9.8	6.31	5.6	5.76	5.12	5.11	5.61	5.42	5.25	5.09	0.03		
Low	18.11	15.83	16.36	16.03	14.06	12.59	13.06	10.76	10.25	10.27	8.02	7.23	−0.94	−.43	−0.43
	7.34	6.83	7.27	7.39	6.5	6.51	6.27	5.76	6.32	5.93	5.9	5.31	0.03		
*A group of students boycotted me and did not want to talk or play with me*															
High	5.76	5.51	4.86	4.51	3.99	3.73	3.74	3.19	3.46	2.9	2.73	2.81	−0.28	−.34	−0.19
	3.54	3.23	3.02	2.66	2.82	2.38	2.83	1.96	1.99	1.82	1.85	1.94	0.01		
Medium	7.67	7.45	7	6.8	5.58	5.2	5.57	4.85	5	3.98	3.51	3.14	−0.41	−.29	−0.23
	5.81	4.99	4.93	5.49	4.56	4.63	4.57	3.71	4.08	3.31	3.29	3.14	0.02		
Low	13.05	13.47	11.45	12.61	10.54	10.4	10.21	8.69	8.08	8.23	5.58	5.43	−0.71	−.30	−0.31
	8.61	8.94	7.96	8.61	7.35	7.18	7.25	6.46	6.75	7.16	5.12	4.82	0.04		
*A student cursed at me to hurt my feelings*															
High	–	39.17	35.38	35.34	27.86	27.93	27.31	28.04	27.1	25.65	23.79	24.32	−1.4	−.41	0.77
	–	10.24	9.31	9.59	8.71	9.64	9.83	9.25	9.71	9.57	8.89	8.77	0.06		
Medium	–	36.19	32.57	33.89	25.57	27.21	25.85	26.19	25.44	24.36	21.5	20.37	−1.39	−.34	0.56
	–	13.2	12.69	11.85	11.31	11	11.57	12.04	12.74	11.93	11.05	11.63	0.06		
Low	–	27.32	29.57	25.3	23.54	20.83	22.97	20.93	19.29	18.67	15.39	14.66	−1.35	−.32	0.42
	–	13.76	13.5	13.24	11.93	11.49	11.61	12.27	12.16	11.85	11.04	10.39	0.07		

**Note**. Missing values indicate that item was not asked that year.

**Table 3.** Means, standard deviations, betas, standard errors, and effect sizes of school climate by cultural groups and year.

	2008	2009	2010	2011	2012	2013	2014	2015	2016	2017	2018	2019			
*N*	1,088	1,058	1,107	1,093	1,149	1,121	1,185	751	798	839	790	820			
	*M* (*SD*)	*M* (*SD*)	*M* (*SD*)	*M* (*SD*)	*M* (*SD*)	*M* (*SD*)	*M* (*SD*)	*M* (*SD*)	*M* (*SD*)	*M* (*SD*)	*M* (*SD*)	*M* (*SD*)	*B* (*SE*)	β	*d*
*Appropriate student class behavior*															
Overall	34.30	34.59	35.80	39.91	41.46	43.11	41.28	42.85	44.27	44.60	48.61	49.35	1.32	.26	0.87
	13.40	13.37	14.20	15.27	15.33	16.21	16.50	17.13	19.11	19.48	20.19	20.31	0.05		
Jews	32.63	32.29	33.62	37.18	39.92	40.83	39.78	39.78	40.83	40.99	43.53	44.89	1.08	.23	0.77
	12.85	12.16	12.72	13.61	14.82	15.64	16.35	15.87	17.99	18.03	17.38	18.33	0.05		
Arabs	38.87	40.97	42.11	47.60	45.92	49.81	46.02	52.12	53.69	54.62	61.51	59.86	1.90	.35	1.18
	13.83	14.48	16.22	16.99	15.92	16.00	16.10	17.49	18.98	19.91	21.09	20.90	0.09		
Secular Jews	31.27	30.68	31.78	34.71	37.34	37.93	36.86	36.86	37.32	38.17	40.47	41.78	0.92	.21	0.69
	12.91	12.05	12.08	12.19	13.68	14.04	14.24	13.67	16.07	16.81	15.55	17.39	0.06		
Religious Jews	35.38	35.42	37.10	42.02	44.89	46.37	45.13	45.80	47.20	46.42	49.47	50.53	1.37	.27	0.96
	12.33	11.77	13.20	14.92	15.72	17.02	18.54	18.29	19.51	19.02	19.20	18.71	0.09		
*Student – teacher caring*															
Overall	55.71	54.66	56.58	59.34	61.36	62.39	62.99	60.84	62.12	62.50	64.37	65.82	0.90	.19	0.64
	13.97	14.02	14.52	14.79	14.60	14.80	15.54	15.56	17.10	17.22	17.67	17.37	0.04		
Jews	53.21	51.78	53.49	56.28	58.74	59.51	60.12	57.33	58.06	59.19	59.89	61.76	0.77	.17	0.56
	13.76	13.68	13.98	14.47	14.55	14.62	15.63	15.07	16.58	17.00	16.52	16.82	0.05		
Arabs	62.53	62.68	65.51	67.95	68.91	70.84	72.03	71.42	73.27	71.70	75.75	75.42	1.20	.31	0.95
	12.17	11.65	12.20	12.06	11.89	11.80	11.20	11.79	13.09	14.25	15.23	14.71	0.07		
Secular Jews	54.42	52.94	54.58	57.04	59.71	60.11	60.71	57.96	58.29	59.32	59.85	61.56	0.64	.14	0.46
	13.85	14.07	14.08	13.90	14.15	13.94	14.66	14.10	15.73	16.35	16.03	16.86	0.06		
Religious Jews	50.77	49.50	51.36	54.81	56.88	58.35	59.04	56.07	57.66	59.08	59.90	62.09	1.02	.21	0.75
	13.30	12.60	13.57	15.45	15.15	15.79	17.29	16.89	18.05	18.21	17.59	16.82	0.09		
*Efforts to prevent violence*															
Overall	68.94	69.25	70.41	71.55	72.05	72.56	73.31	73.99	74.31	74.31	75.27	75.96	0.63	.14	0.45
	15.72	14.43	14.55	14.86	14.70	14.91	14.93	14.85	15.63	15.88	15.68	15.44	0.04		
Jews	69.11	69.30	69.83	71.21	71.50	71.80	72.73	72.94	72.69	72.84	72.99	73.71	0.42	.09	0.28
	16.85	15.81	15.75	16.07	15.88	16.04	16.29	15.92	16.67	17.07	16.42	16.45	0.05		
Arabs	68.47	69.11	72.09	72.53	73.65	74.79	75.13	77.15	78.75	78.37	81.05	81.25	1.17	.35	1.10
	12.12	9.61	10.18	10.71	10.41	10.63	9.22	10.44	11.20	11.03	11.80	11.06	0.06		
Secular Jews	72.73	72.51	73.31	74.09	74.19	74.58	75.27	75.63	74.75	74.94	74.75	74.83	0.23	.06	0.14
	14.44	13.49	13.37	13.28	13.89	13.33	13.49	13.13	14.45	14.49	14.27	15.33	0.05		
Religious Jews	61.71	63.03	63.06	65.57	66.24	66.49	68.03	67.47	68.98	68.99	69.39	71.71	0.82	.14	0.54
	18.90	18.00	17.78	19.28	18.11	19.18	19.73	19.42	19.58	20.56	19.70	18.23	0.10		
*Feedback to promote learning*															
Overall	64.38	64.67	65.57	66.78	67.97	69.12	67.80	68.75	69.25	69.60	70.37	71.53	0.60	.14	0.47
	13.57	13.15	13.51	14.15	13.99	14.09	14.79	14.61	15.92	16.26	17.02	16.88	0.04		
Jews	62.56	62.50	63.00	63.91	65.27	66.20	65.00	65.57	65.55	66.32	66.00	67.39	0.41	.09	0.32
	13.81	13.23	13.27	13.84	13.90	13.78	14.79	14.14	15.49	16.03	16.18	16.34	0.05		
Arabs	69.34	70.70	72.96	74.88	75.77	77.65	76.58	78.35	79.41	78.70	81.48	81.30	1.05	.29	0.94
	11.55	10.87	11.32	11.65	11.03	11.24	10.88	11.47	12.29	13.14	13.77	13.85	0.06		
Secular Jews	63.88	63.79	64.38	64.62	65.95	66.45	65.33	65.91	65.58	66.19	65.57	66.76	0.23	.05	0.19
	13.60	12.90	12.89	13.08	13.57	13.04	13.75	13.26	14.78	15.34	15.65	16.35	0.05		
Religious Jews	59.86	60.00	60.28	62.52	63.92	65.73	64.42	64.92	65.49	66.68	66.75	68.50	0.77	.16	0.57
	13.88	13.53	13.62	15.17	14.49	15.11	16.57	15.85	16.72	17.29	17.26	16.35	0.08		
*Peer relations*															
Overall	65.04	65.34	66.36	68.94	70.12	71.10	70.25	70.07	71.76	72.19	74.25	76.88	0.93	.26	1.05
	9.99	10.31	10.32	10.79	10.91	11.17	11.96	12.26	13.05	12.91	13.22	12.45	0.03		
Jews	63.97	63.69	64.46	66.99	68.22	69.43	68.50	67.65	69.29	70.28	70.91	74.39	0.80	.23	0.93
	9.94	9.92	9.84	10.26	10.72	10.89	12.10	11.76	12.81	12.71	12.48	12.27	0.04		
Arabs	67.96	69.92	71.82	74.44	75.59	75.98	75.75	77.37	78.54	77.49	82.75	82.75	1.21	.37	1.45
	9.56	9.99	9.73	10.36	9.56	10.52	9.65	10.77	11.17	11.97	11.08	10.80	0.05		
Secular Jews	62.80	62.12	63.49	65.48	66.42	67.30	66.19	65.07	66.35	68.01	68.05	71.56	0.63	.19	0.78
	10.03	9.40	9.66	9.35	10.14	9.83	11.24	10.84	12.16	12.20	11.89	12.22	0.04		
Religious Jews	66.36	66.76	66.30	69.95	71.71	73.52	72.79	72.96	74.62	74.70	76.50	79.53	1.12	.31	1.32
	9.34	10.19	9.96	11.28	10.96	11.65	12.47	11.87	12.25	12.42	11.65	10.61	0.06		
*Feeling unsafe*															
Overall	9.21	8.91	8.60	7.58	6.68	6.20	6.37	6.02	5.75	5.49	4.53	4.41	−0.44	−.28	−0.94
	6.48	6.17	5.77	5.19	4.84	4.66	5.20	4.44	4.91	4.61	3.10	3.23	0.01		
Jews	6.97	6.70	6.78	6.02	5.23	4.91	4.74	4.75	4.45	4.30	4.11	3.85	−0.30	−.32	−1.00
	3.60	3.51	3.37	3.14	2.80	2.95	2.96	2.62	2.70	2.52	2.48	2.52	0.01		
Arabs	15.31	15.06	13.83	11.97	10.85	9.97	11.47	9.87	9.29	8.80	5.60	5.72	−0.84	−.39	−1.44
	8.41	7.63	7.75	7.01	6.71	6.40	7.09	6.22	7.30	6.92	4.11	4.20	0.04		
Secular Jews	6.70	6.52	6.63	6.06	5.33	5.14	5.01	4.97	4.78	4.52	4.42	4.16	−0.25	−.29	−0.88
	3.19	2.92	2.89	2.89	2.60	2.73	2.83	2.46	2.58	2.47	2.49	2.52	0.11		
Religious Jews	7.52	7.04	7.07	5.95	5.03	4.47	4.26	4.27	3.87	3.84	3.50	3.29	−0.41	−.37	−1.22
	4.27	4.41	4.13	3.59	3.15	3.28	3.16	2.87	2.82	2.54	2.36	2.43	0.02		
*Belongingness*															
Overall	69.71	69.82	70.28	72.42	74.07	74.63	73.76	73.32	74.72	75.40	76.75	77.85	0.69	.20	0.70
	11.70	11.18	11.37	11.31	10.70	11.20	11.66	11.75	12.17	12.19	12.09	11.59	0.03		
Jews	69.87	69.65	69.55	71.79	73.66	73.96	73.45	72.17	73.46	74.86	74.88	76.58	0.57	.16	0.58
	11.78	11.33	11.51	11.56	10.87	11.08	11.92	11.81	12.16	11.99	11.68	11.19	0.04		
Arabs	69.29	70.31	72.40	74.19	75.26	76.59	74.75	76.78	78.18	76.89	81.50	80.85	0.99	.29	0.98
	11.51	10.75	10.71	10.39	10.14	11.33	10.75	10.90	11.53	12.62	11.83	11.98	0.06		
Secular Jews	72.08	72.00	71.90	73.56	75.31	75.26	74.76	73.46	74.55	75.82	75.24	76.98	0.39	.13	0.46
	10.71	9.47	9.91	9.11	9.37	9.30	10.10	9.99	10.60	10.82	10.32	10.56	0.04		
Religious Jews	65.36	65.06	64.93	68.34	70.50	71.49	71.05	69.57	71.48	73.18	74.06	75.81	0.94	.22	0.84
	12.58	13.13	12.96	14.67	12.72	13.54	14.44	14.56	14.39	13.73	14.06	12.27	0.07		

**Table 4.** Means, standard deviations, betas, and effect sizes of school climate by socioeconomic status and year.

	2008	2009	2010	2011	2012	2013	2014	2015	2016	2017	2018	2019			
*N*	1,088	1,058	1,107	1,093	1,149	1,121	1,185	751	798	839	790	820			
	*M* (*SD*)	*M* (*SD*)	*M* (*SD*)	*M* (*SD*)	*M* (*SD*)	*M* (*SD*)	*M* (*SD*)	*M* (*SD*)	*M* (*SD*)	*M* (*SD*)	*M* (*SD*)	*M* (*SD*)	*B* (*SE*)	β	*d*
*Appropriate students’ class behavior*															
High	32.04	30.07	33.11	35.34	38.55	39.01	38.92	38.47	38.14	38.52	41.54	38.5	0.81	.20	0.16
	11.77	10.83	11.11	11.54	12.1	13.13	14.52	13.22	15.16	15	15.33	14.08	0.07		
Medium	34.71	34.59	35.22	39.2	42.39	42.69	41.94	43.59	44.58	44.92	47.31	50.27	1.34	.26	0.32
	13.6	13.14	13.49	14.45	15.88	16.76	16.59	17.4	19.33	20.78	19.72	20.04	0.07		
Low	35.7	38.71	38.62	44.79	42.91	47.27	42.43	46.26	49.45	49.5	56.94	58.31	1.78	.32	0.45
	14.12	13.97	16.5	17.33	16.6	16.76	17.49	19.09	20.43	19.79	21.87	20.94	0.09		
*Student – teacher caring*															
High	52.94	48.9	52.96	53.59	57.29	57.15	59.37	55.26	56.66	56.66	57.79	57.57	0.61	.14	0.11
	13.65	13.04	13.98	13.05	13.55	13.11	15.06	13.95	15.12	16.11	15.77	14.99	0.08		
Medium	54.72	54.32	54.92	59.04	60.78	61.81	62.25	60.88	60.16	62.54	63.16	65.35	0.92	.20	0.23
	13.77	13.85	13.79	14.59	14.78	14.99	15.67	15.86	17.17	17.64	17.49	17.3	0.07		
Low	59.47	60.49	61.85	64.95	65.7	68.11	67.21	65.94	69.66	67.5	72.12	74.21	1.16	.26	0.34
	13.46	12.55	14.53	14.3	13.92	13.59	14.66	15.02	15.95	15.96	16.71	15.66	0.07		
*Efforts to prevent violence*															
High	69.24	67.91	69.16	69.59	70.26	70.17	72.19	71.98	71.84	70.63	72.58	71.14	0.33	.07	0.04
	16.48	15.1	15.74	15.35	15.64	15.04	15.84	15.21	16.17	16.96	15.26	15.29	0.08		
Medium	68.66	69.92	69.82	72.4	71.93	73	73.17	74.15	73.16	74.74	74.11	75.6	0.57	.12	0.16
	15.99	15.13	14.77	15.13	15.07	15.44	15.79	15.9	16.39	16.3	16.48	16.31	0.07		
Low	69.92	69.7	72.31	72.54	74.06	74.57	74.52	75.87	78.01	76.89	79.23	80.95	0.94	.23	0.28
	13.85	12.8	12.96	13.56	12.82	13.2	12.75	12.86	13.28	13.62	14.33	12.66	0.06		
*Feedback to promote learning*															
High	61.01	58.55	60.54	59.85	61.92	62.15	62.64	62.08	62.57	62.13	62.38	62.25	0.27	.07	0.03
	13.74	12.68	12.92	12.29	13.3	12.22	14.25	13.28	14.56	15.37	15.72	14.96	0.07		
Medium	63.68	64.78	64.33	66.57	67.45	68.82	67.02	69.07	67.77	69.61	69.49	71.24	0.61	.14	0.17
	13.39	12.9	12.83	13.89	13.62	13.83	14.61	14.36	15.77	16.23	16.36	16.63	0.06		
Low	68.11	70.03	71.19	73.21	73.56	75.99	73.24	74.54	77.22	76.06	79.1	80.64	0.95	.24	0.31
	12.39	11.25	12.63	12.85	12.66	12.21	13.37	13.59	13.6	14.08	14.85	13.82	0.06		
*Peer relations*															
High	64.55	63.64	65.5	67.32	68.67	69.6	69.57	68.33	69.46	70.41	70.69	72.71	0.70	.24	0.24
	9.27	8.9	8.33	8.93	9.31	8.84	10.31	9.62	10.69	10.82	11.01	10.03	0.05		
Medium	65.7	65.32	66.04	68.17	70.85	70.57	70.59	70.2	71.76	72.35	74.04	77.24	0.93	.05	0.30
	9.68	10.82	9.81	10.66	10.7	11.56	11.86	12.63	12.9	13.6	13.22	12.62	0.05		
Low	64.89	67.4	67.63	71.63	70.75	73.27	70.51	71.62	73.79	73.59	77.93	80.31	1.10	.28	0.39
	10.7	10.34	11.92	11.67	12.03	11.84	13.18	13.72	14.74	13.48	14.2	13.15	0.06		
Feeling unsafe															
High	5.8	5.88	5.94	5.29	4.54	4.35	4.16	4.23	4.12	3.78	3.72	3.76	−0.28	−.30	−0.13
	2.81	2.84	2.89	2.66	2.21	2.51	2.38	2.14	2.16	1.99	2.04	2.18	0.01		
Medium	8.07	7.46	7.55	6.57	5.78	5.31	5.35	5	4.83	4.56	4.06	3.94	−0.38	−.34	−0.23
	4.52	4.4	4.11	3.74	3.62	3.41	3.7	2.88	3.19	2.8	2.47	2.78	0.02		
Low	13.52	13.53	12.05	10.85	9.46	9.05	9.58	8.85	8.42	8.17	5.87	5.64	−0.69	−.33	−0.35
	8.37	7.66	7.53	6.63	6.29	6.03	6.91	5.89	7.06	6.42	4.05	4.15	0.03		
*Belongingness*															
High	72.41	70.25	71.82	72.54	75.06	74.82	75.48	73.18	75.26	75.26	75.08	75.83	0.41	.14	0.10
	10.08	9.39	9.7	9.26	9.25	8.61	10.44	9.96	9.63	10.38	10.16	9.07	0.05		
Medium	69.36	69.87	69.14	72.39	73.86	74.25	73.32	73.41	72.97	75.54	75.8	77.03	0.65	.18	0.20
	11.99	11.83	11.51	11.12	11.09	11.64	11.98	12.41	13.06	12.74	12.71	12.44	0.05		
Low	68.37	69.64	70.64	72.68	73.94	74.97	72.98	73.41	76.44	75.26	79.48	80.84	0.95	.25	0.33
	11.58	11.49	12.17	12.65	10.89	12.43	12.02	12.47	12.8	12.89	12.61	11.97	0.06		

## Data Availability

Data will be made available in response to reasonable requests.
